# Heart failure of very rare aetiology—haemochromatosis Type 3: a case report

**DOI:** 10.1093/ehjcr/ytae637

**Published:** 2024-11-29

**Authors:** Aistė Montvilaitė-Laurinavičienė, Rūta Dirsienė, Gintarė Neverauskaitė-Piliponienė, Audra Banišauskaitė, Marius Šukys, Gintarė Šakalytė, Eglė Ereminienė

**Affiliations:** Faculty of Medicine, Lithuanian University of Health Sciences, A. Mickevičiaus Str. 9, 44307 Kaunas, Lithuania; Faculty of Medicine, Lithuanian University of Health Sciences, A. Mickevičiaus Str. 9, 44307 Kaunas, Lithuania; Department of Cardiology, Lithuanian University of Health Sciences, Eivenių Str. 2, 50161 Kaunas, Lithuania; Faculty of Medicine, Lithuanian University of Health Sciences, A. Mickevičiaus Str. 9, 44307 Kaunas, Lithuania; Department of Cardiology, Lithuanian University of Health Sciences, Eivenių Str. 2, 50161 Kaunas, Lithuania; Faculty of Medicine, Lithuanian University of Health Sciences, A. Mickevičiaus Str. 9, 44307 Kaunas, Lithuania; Department of Radiology, Lithuanian University of Health Sciences, Eivenių Str. 2, 50161 Kaunas, Lithuania; Faculty of Medicine, Lithuanian University of Health Sciences, A. Mickevičiaus Str. 9, 44307 Kaunas, Lithuania; Department of Genetics and Laboratory Medicine, Lithuanian University of Health Sciences, Eivenių Str. 2, 50161 Kaunas, Lithuania; Faculty of Medicine, Lithuanian University of Health Sciences, A. Mickevičiaus Str. 9, 44307 Kaunas, Lithuania; Department of Cardiology, Lithuanian University of Health Sciences, Eivenių Str. 2, 50161 Kaunas, Lithuania; Institute of Cardiology, Lithuanian University of Health Sciences, Sukilėlių Av. 15, 50161 Kaunas, Lithuania; Faculty of Medicine, Lithuanian University of Health Sciences, A. Mickevičiaus Str. 9, 44307 Kaunas, Lithuania; Department of Cardiology, Lithuanian University of Health Sciences, Eivenių Str. 2, 50161 Kaunas, Lithuania; Institute of Cardiology, Lithuanian University of Health Sciences, Sukilėlių Av. 15, 50161 Kaunas, Lithuania

**Keywords:** Haemochromatosis type 3, Heart failure, Dilated cardiomyopathy, Rare disease, Case report

## Abstract

**Background:**

Haemochromatosis is a pathological condition characterized by the accumulation of iron in parenchymal organs, leading to toxic damage and dysfunction. Cardiac haemochromatosis represents one of the rare causes of severe heart failure (HF) that can be potentially prevented with targeted treatment.

**Case Summary:**

We present the case of a 41-year-old female who was hospitalized for decompensated HF. Echocardiography revealed severe systolic dysfunction with a phenotype of dilated cardiomyopathy, accompanied by secondary moderate mitral regurgitation and severe tricuspid regurgitation (TR). To differentiate potential causes of HF, coronary angiography, cardiac magnetic resonance imaging (MRI), and endomyocardial biopsy were performed. Based on clinical findings, laboratory results, cardiac MRI, and endomyocardial biopsy data, a diagnosis of haemochromatosis was confirmed, and mutations in the *TFR2* gene, responsible for haemochromatosis Type 3, were identified. The patient was treated in accordance with the latest European Society of Cardiology HF guidelines, and specific treatment for haemochromatosis, including therapeutic phlebotomy and iron chelation therapy, was initiated, resulting in a significant positive outcome.

**Discussion:**

Investigating the aetiology of HF is essential, as even rare causes can be identified, and specific treatments are available that significantly improve prognosis and survival.

**Learning points**

- Haemochromatosis Type 3 is classified as a very rare disease and confirmation of its diagnosis requires the involvement of a multidisciplinary team and a wide range of investigations.
- Genetic counselling, detailed genetic testing, and determination of the exact cause of cardiomyopathy have a significant impact on choosing the most appropriate treatment tactics and are very important for achieving the best prognosis.

## Introduction

Dilated cardiomyopathy (DCM) is characterized by left ventricular (LV) dilation with systolic dysfunction in the absence of abnormal loading conditions (e.g. congenital heart disease, valvular disease) or significant coronary artery disease.^1^ Dilated cardiomyopathy encompasses both genetic and acquired disorders, with a prevalence of approximately 1 in 2500 people.^2^ The diverse aetiology of DCM necessitates a thorough diagnostic work-up to address the underlying pathology.

Haemochromatosis is a clinical syndrome marked by the pathological accumulation of iron in parenchymal liver cells, the heart, and endocrine glands, leading to organ damage and dysfunction.^3,4^ Early diagnosis and timely initiation of appropriate treatment are crucial for preventing irreversible organ damage.

Cardiac haemochromatosis, also known as primary iron overload cardiomyopathy, is one of the rare causes of severe heart failure (HF) that can potentially be prevented with specific interventions. Haemochromatosis type 3 is classified as a very rare disease, with a prevalence of less than 1 in 1 000 000 people.^5^ Fewer than 50 cases of type 3 haemochromatosis have been reported in the literature.^5^

We present the case of a 41-year-old female diagnosed with haemochromatosis type 3, which manifested as severe HF and was effectively managed through phlebotomy, iron chelation therapy, and optimal HF treatment.

## Summary figure

**Figure:**
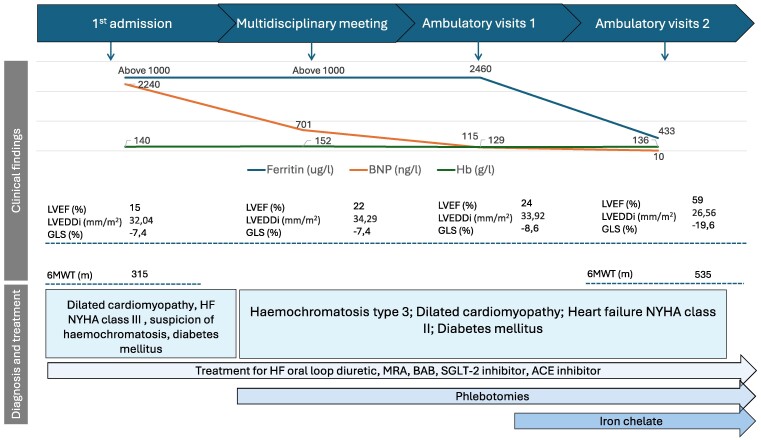


Timeline of events depicting the clinical data of the patient's diagnosis, treatment, and the results of imaging and laboratory tests.

## Case presentation

A 41-year-old female arrived to the emergency department with progressive shortness of breath during mild exertion and occasionally at rest, along with abdominal distension, leg swelling, and decreased urine output. Over the past 6 months, she had been treated twice at a secondary-level hospital for exacerbations of HF, with echocardiographic findings indicating a reduced left ventricular ejection fraction (LVEF) of 30%. Unfortunately, there was no clinical improvement observed with the HF medications she was receiving (spironolactone 25 mg ×1, torasemide 10 mg ×1, ivabradine 5 mg ×2). The patient had no other known chronic illnesses, addictions, or significant risk factors. Laboratory results obtained in the emergency department revealed a moderate increase in troponin I (0.16 μg/L; normal value < 0.04 μg/L), a significant elevation in brain natriuretic peptide (BNP) (2240.7 ng/L; normal value 0–35 ng/L), and increased D-dimer levels (12.1 mg/L; normal value 0–0.5 mg/L); additionally, she presented with hypomagnesaemia, hypernatremia, normal renal function, and a normal complete blood count (*Table 1*).

**Table 1:** Laboratory results

	2021 09	2021 12	2022 04	2022 12	2023 04	2024 02
BNP (ng/L)	2240.7	701.2	157.5	21.7	10.2	9.7
Alanine aminotransferase (IU/L)	138	63	72	123	37	
Aspartic aminotransferase (IU/L)	217	82	72	84	42	
Gammaglutamyltransferase (IU/L)	97	92	61			
Alkaline phosphatase (IU/L)	81	72	81			
Total bilirubin (μmol/L)	19.95	16.25	11.9	13.9		
INR	1.6	1.4	1.2			
Albumin (g/L)	41					
Troponin (μg//L)	0.16		0.04			
Haemoglobin (g/L)	140	136	130	130	136	137
Haematocrit (%)	41.3	38.4	37.1	38.2	38.8	40.0
Platelets (×10×9/L)	168–164	107	111	141	145	137
Ferritin (μg/L; norm 15–150)	>1000 (unspecified)	3945	2410.5	1367.5	433.9	135
Transferrin saturation (%; norm 15–45)	110	92	86	92		
Iron (μmol/L, norm 10.7–32.2)	37.6	49.7	48.3	55.2		56.2
Uric acid (mmol/L)	650	459	291			261
Glucose (mmol/L)	8.74		7.44		7.35	
Creatinine (mmol/L)	66	61	48	51		52

A chest X-ray performed in the emergency department showed right-sided hydrothorax, moderate congestive changes, and dilated cardiac borders. To further investigate the cause of her shortness of breath, a chest computed tomography scan was conducted, which revealed dilated cardiac cavities, reflux of contrast material into the hepatic veins, mediastinal lymphadenopathy, and right hydrothorax, but no signs of pulmonary embolism were identified. Abdominal ultrasound revealed the presence of ascites.

The patient was subsequently admitted to the Heart Failure Unit of the Department of Cardiology for evaluation of cardiomyopathy of unknown origin and decompensated HF.

**Figure 1:**
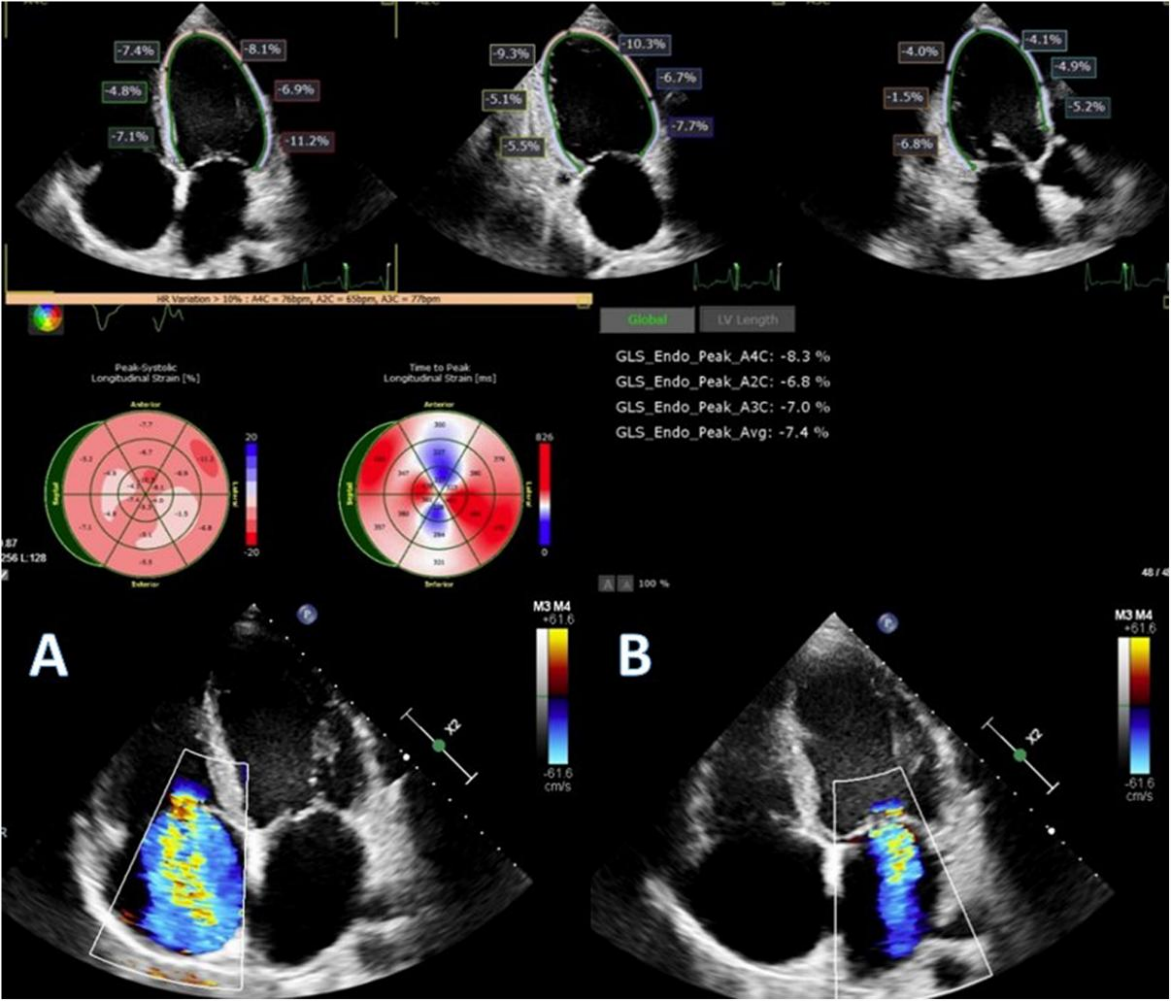
2D echocardiography: left ventricular longitudinal strain measurement (apical position four-chamber, two-chamber, and three-chamber views; strain values in the middle ‘bull’s-eye’ view). (*A*) Four-chamber view of the apical position: dilatation of all cardiac chambers, secondary large tricuspid regurgitation by colour Doppler; (*B*) apical position four-chamber view: colour Doppler—secondary moderate mitral regurgitation.

Echocardiography demonstrated markedly reduced LV systolic function [EF 15%, global longitudinal strain (GLS) −7.4], consistent with a DCM phenotype (LV end-diastolic size index 32.04 mm/m^2^). There was also right ventricular dysfunction (tricuspid annular plane systolic excursion 16 mm, *S*′ 9.5 cm/s, fractional area change 21.4%), secondary moderate mitral regurgitation, and significant tricuspid regurgitation (TR) (*Figure 1*).

Coronary angiography was performed to differentiate the potential causes of systolic dysfunction and revealed no stenosis.

Several episodes of non-sustained ventricular tachycardia (VT) were recorded during Holter monitoring, and given the severely advanced HF of unknown aetiology, an implantable cardioverter-defibrillator (ICD) was placed based on a multidisciplinary consensus.

**Figure 2:**
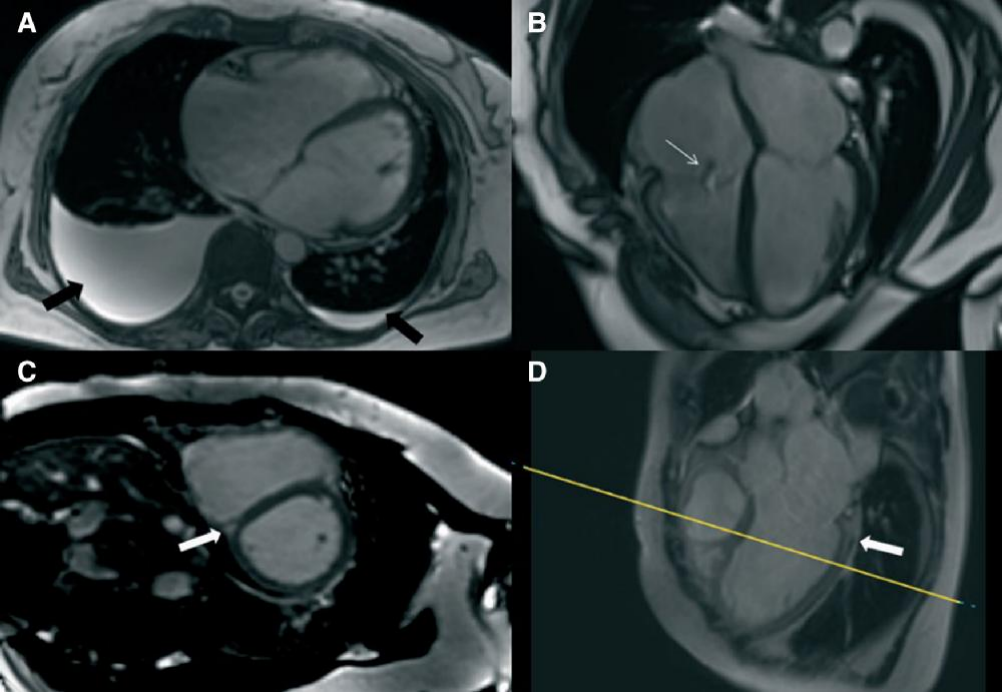
Cardiac magnetic resonance imaging: (*A*) bilateral hydrothorax; (*B*) dilated cardiac chambers, biventricular dysfunction: left ventricular ejection fraction 22%; right ventricular ejection fraction 32%; (*C*, *D*) signs of contrast material accumulation at the point of insertion of the ventricles.

**Figure 3:**
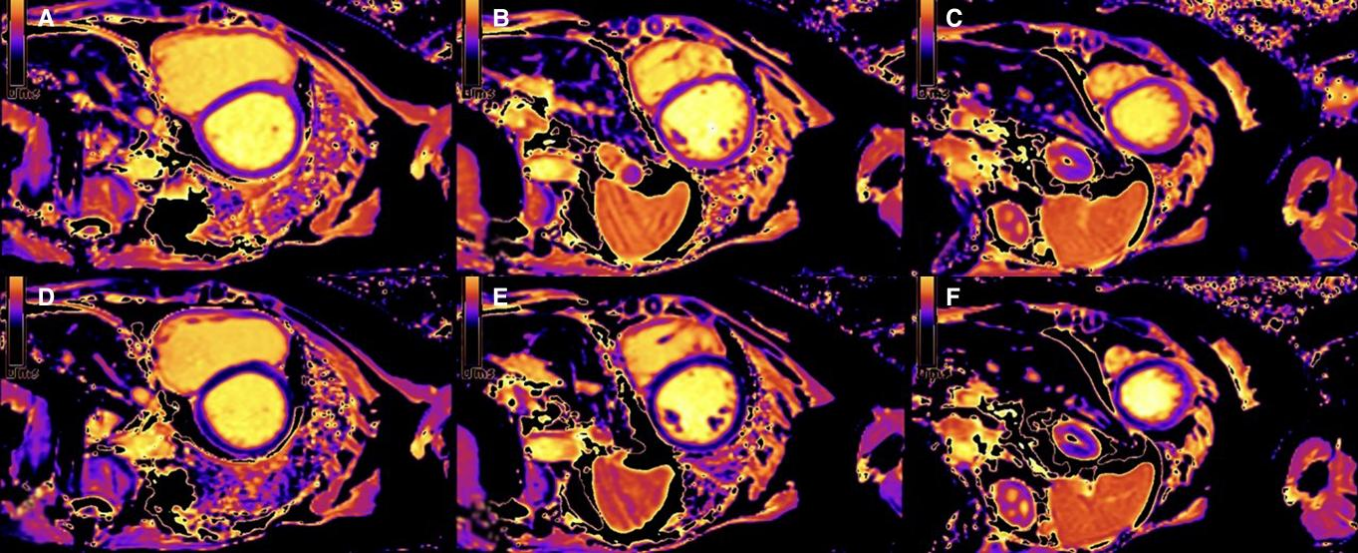
Cardiac magnetic resonance imaging: sequence of T1 maps, short-axis images. (*A–C*) in diastole with values of relaxation times (tables), (*D–F*) in systole.

**Figure 4:**
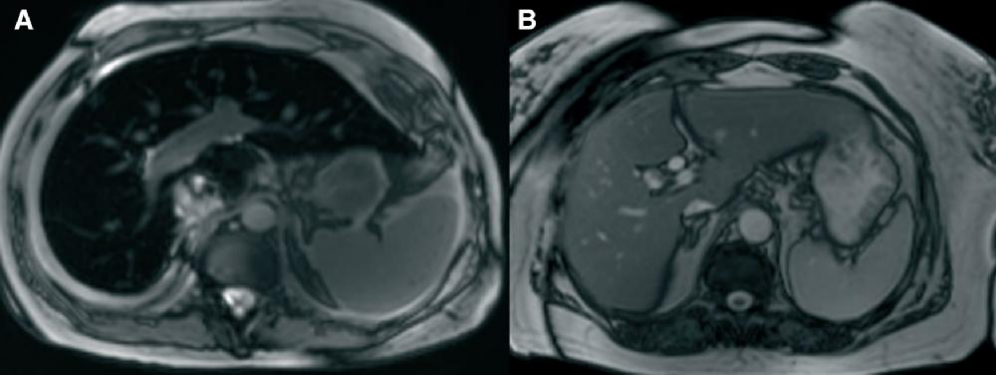
Abdominal magnetic resonance imaging: (*A*) liver with diffusely very low signal intensity; (*B*) liver with normal signal intensity.

For differential diagnosis, cardiac magnetic resonance imaging (MRI) was performed, which showed dilated ventricles with significantly reduced biventricular systolic function (LVEF 22%, RVEF 32%), and both atria were dilated (*Figure 2*). Significantly shortened myocardial T1 and T2 relaxation times were observed [T1 800–850 ms (normal ∼1200 ms), T2 20–25 ms (normal 37–44 ms)], along with non-specific fibrosis at the lower ventricular junction point (*Figures 2* and *3*). Abdominal MRI revealed a liver with very low signal intensity (*Figure 4*). In summary, the MRI findings were characteristic of the DCM phenotype, leading to a suspected haemochromatosis.

Considering the possibility of haemochromatosis, ferritin and transferrin saturation tests were performed, revealing markedly elevated ferritin levels (3945 ng/mL) and transferrin saturation (110%). The patient had a consultation with a geneticist, where initial genetic testing was negative, indicating no common variants for the *HFE* gene [(NM_000410.3) c.845G > A p.C282Y and c.187C > G p.H63D], which is commonly associated with hereditary haemochromatosis. Subsequently, a second-line genetic test utilizing next-generation sequencing identified alterations in the *TFR2* gene, which are responsible for haemochromatosis Type 3. To further clarify the aetiology of the myocardial damage, an endomyocardial biopsy was performed. Following a thorough review of the biopsy findings with a pathologist, iron accumulation was observed in the myocytes. This led to decision to perform exon sequencing with analysis on virtual panel of rare types of haemochromatosis. Two variants in *TRF2* (NM_003227.4) gene (confirmed transposition) were found: c.1288G > A p.(Gly430Arg) and c.287-3C > G. 1288G > A variant is extremely rare in control populations, predicted as damaging *in silico* tools, several times mentioned in literature as causative for haemochromatosis. Variant c.287-3C>G is also extremely rare in control populations but not mentioned in literature or Clinvar. Based on this data, it was concluded that *TRF2* gene variants are the most likely causative for phenotype, thus haemochromatosis type 3 was diagnosed.

A multidisciplinary council confirmed the diagnosis of type 3 haemochromatosis with involvement of the heart, liver, and pancreas. Consequently, a combined treatment approach was initiated, incorporating phlebotomy and optimal pharmacological management of HF with carvedilol, perindopril, empagliflozin, spironolactone, and torasemide, in accordance with the latest ESC recommendations.^6^ Due to insufficient response to phlebotomy, characterized by persistently elevated ferritin levels and significant iron overload cardiomyopathy, iron chelation therapy with deferiprone was initiated in November 2022.

Throughout the course of combined treatment, a positive clinical effect was achieved, with complete regression of dyspnoea, absence of congestion, and improved physical performance, as evidenced by a 6-minute walk test result of 535 m. A significant decrease in ferritin and BNP levels, along with improvements in other laboratory tests, was observed during treatment (*Table 1*). Normal LV systolic function (LVEF 55%, GLS −25%) was achieved at the last examination. Additionally, there was a significant reduction in both mitral and TR following appropriate treatment. The patient is regularly monitored by a cardiologist, endocrinologist, and gastroenterologist. Treatment with carvedilol, metformin, and empagliflozin continues, along with phlebotomy to maintain ferritin levels below 150 μg/L.

## Discussion

We presented a case of DCM caused by a very rare aetiology—haemochromatosis Type 3. An additional extended gene study using a new DNA sequencing method identified the underlying cause of DCM and facilitated the selection of specific treatments, including iron chelation and phlebotomies, which significantly altered the clinical course and prognosis for our patient.

Dilated cardiomyopathy is one of the leading causes of HF^1^ with a prevalence of 5–8 per 100 000 people, predominantly affecting younger adults.^7^ Clinical manifestations can vary from uncomplicated cases to extremely severe and rapidly deteriorating conditions.^8^

Genetic testing is essential in diagnosing DCM, as identifying pathogenic gene variants allows for targeted treatment, prevention of potential complications, and improved prognostic information for family members. Over 50 genes associated with DCM have been identified, with strong scientific evidence supporting the involvement of 12 specific genes in disease development. These include *BAG3*, *DES*, *FLNC*, *LMNA*, *MYH7*, *PLN*, *RBM20*, *SCN5A*, *TNNC1*, *TNNT2*, *TTN*, and *DSP*.^7^ A comprehensive family history is vital for assessing the likelihood of a genetic aetiology and for identifying potential genetic syndromes associated with DCM.^8^

When diagnosing DCM and determining appropriate diagnostic and treatment strategies, it is crucial to discuss each case with a multidisciplinary team that includes cardiologists, geneticists, radiologists, and other specialists. In the presented case, the patient did not exhibit gene mutations typically associated with DCM, together with clinical findings, suggesting the possibility of an alternative genetic disorder as the underlying aetiology.

Haemochromatosis Type 3 is classified as a very rare disease, with the pathogenic allele frequency of *TFR2* hereditary haemochromatosis estimated at approximately 0.00008 to 0.0002. In our case, initial genetic testing caused some confusion, as no mutations were detected in the *HFE* gene, which is responsible for the most common forms of haemochromatosis. However, a diagnosis was strongly suspected based on MRI and laboratory findings. Subsequent genetic testing confirmed our suspicion and allowed us to diagnose the very rare type 3 haemochromatosis.

Haemochromatosis is characterized by transferrin saturation greater than 55% and serum ferritin concentrations exceeding 200 μg/L in women and 300 μg/L in men. It is important to note that serum ferritin levels do not always correlate with cardiomyocyte iron content; thus, further non-invasive tests or biopsies should be conducted if cardiac damage is suspected.^3^

In our patient, haemochromatosis was suspected only after cardiac MRI revealed specific shortening of myocardial T1 and T2 relaxation times and visible infiltration at the liver margin. Ferritin was not tested at the onset of the disease due to the patient’s normal haemoglobin levels, relative youth, and the relatively brief duration of symptoms. The latest ESC guidelines for HF treatment^6^ recommend checking ferritin and transferrin saturation levels in all patients with HF.

Transthoracic echocardiography is the non-invasive first-choice method for assessing potential cardiac damage in patients with iron overload syndrome.^3^ Early signs of cardiac damage include LV diastolic dysfunction with restrictive filling and left atrial dilation. In later stages, there may be dilatation of the cardiac chambers and a decline in ventricular function.^4^ In cases of haemochromatosis, echocardiography is a useful tool for evaluating potential signs of cardiomyopathy; however, this test does not detect iron deposits, necessitating additional assessments.

Cardiac MRI is the only non-invasive method capable of detecting and quantifying iron deposits in the myocardium and liver.^3^ Due to the paramagnetic effect of iron, tissues containing iron deposits exhibit shorter relaxation times in T1, T2, and T2 sequences, as well as low signal intensity in other sequences.^9^ The relaxation time is inversely related to the amount of iron present in the myocardium: greater iron content results in shorter relaxation times.^9^ MRI T2* is a sufficiently sensitive and highly specific sequence for detecting and quantifying myocardial iron deposits, but when iron content is low, T1 relaxation time is more sensitive, though less specific, than T2.^9^

Phlebotomy is the standard treatment for hereditary haemochromatosis and, when initiated early, can prevent irreversible organ damage caused by iron deposition. Unfortunately, due to the rarity of the disease and the challenges associated with diagnosis, many patients remain undiagnosed or receive a diagnosis too late, particularly those with undetectable *HFE* mutations who already exhibit significant organ damage. Phlebotomy effectively reduces myocardial iron deposits and improves LV function, leading to a decrease in size, mass, and left atrial diameter.

Phlebotomy should be initiated when serum ferritin levels exceed 300 µg/L in men and 200 µg/L in women, regardless of symptomatology. Treatment should occur at intervals of at least 1 week until ferritin levels reach 10–20 µg/L. In order to avoid anaemia, haemoglobin levels must be re-evaluated prior to each phlebotomy session. Phlebotomy should be withheld if haematocrit levels fall below 80% of baseline. The standard volume of blood removed is 400–500 mL, adjusted based on the patient's age, body weight, and co-morbidities. The target serum ferritin level is 50–100 µg/L.^4^ By removing excess iron from tissues, phlebotomy reduces free radical production and mitigates organ damage.^4^

In our case, phlebotomy was performed every 1–2 weeks for the initial 16 months and continues periodically every few weeks to months. The patient has experienced no episodes of anaemia, allowing for ongoing phlebotomy based on ferritin concentration. Total blood counts were checked before each therapeutic phlebotomy, and ferritin levels were monitored approximately every month.

Iron chelation therapy is the first-line treatment for patients with anaemia and significant cardiac involvement. Given our patient’s severe cardiac damage, the use of the iron chelator deferiprone was indicated. Chelation therapy was administered for 8 months until ferritin levels fell below 1000 µg/L, as recommended.^4,10^

The combined treatment of phlebotomy, deferiprone, and optimal medical management for HF led to a significant improvement in the patient’s physical tolerance, overall condition, and positive trends in laboratory and echocardiographic parameters. However, the decision to implant an implantable ICD may have been somewhat premature, as a rapid positive response to treatment was observed. After the device was implanted, it became challenging to fully assess the dynamics of MRI data due to artefacts caused by the ICD.

## Conclusion

Haemochromatosis Type 3 is classified as a very rare disease, and its diagnosis necessitates the involvement of a multidisciplinary team along with a comprehensive range of investigations. Genetic counselling, detailed genetic testing, and the identification of the precise cause of cardiomyopathy are crucial for selecting the most appropriate treatment strategies and achieving the best possible prognosis.

## Data Availability

Non-identifiable data underlying this article will be made available upon reasonable request to the corresponding author.

## Lead author biography

Dr Aistė Laurinavičienė earned her MD from Lithuanian University of Health Sciences and finished her cardiology residency program in Lithuanian University of Health Sciences Kaunas Clinics. Her interests include heart failure, imaging, and cardiomyopathies. She attended The London Postgraduate Course in Heart Failure Management (PCHF) 2023/2024.

## Notes

**Consent:** The authors confirm that written consent for the submission and publication of this case report, including images and associated text, has been obtained from the patient in accordance with COPE guidance.
